# WHO Functioning and Disability Disaggregation (FDD11) tool: a reliable approach for disaggregating data by disability

**DOI:** 10.1186/s13690-022-01001-2

**Published:** 2022-12-07

**Authors:** Lindsay Lee, Kaloyan Kamenov, Carolina Fellinghauer, Carla Sabariego, Somnath Chatterji, Alarcos Cieza

**Affiliations:** 1 Sensory Functions, Disability and Rehabilitation, Department of Noncommunicable Diseases, World Health Organization, Avenue Appia 20, 1211 Geneva, Switzerland; 2 Department of Health Sciences and Medicine, University of Lucerne, Lucerne, Switzerland; 3 Swiss Paraplegic Research, Nottwil, Switzerland; 4 Center for Rehabilitation in Global Health Systems, WHO Collaborating Center, University of Lucerne, Lucerne, Switzerland; 5 Department of Data and Analytics, World Health Organization, Geneva, Switzerland

**Keywords:** Functioning and disability disaggregation tool (FDD11), Disability, Functioning, Disaggregation, Psychometric properties

## Abstract

**Background:**

There is a global scarcity of good quality disability data, which has contributed to a lack of political will to address the challenges that persons with disabilities face. The current paper proposes a way forward to overcome this gap by demonstrating the psychometric properties of the World Health Organization Functioning and Disability Disaggregation Tool (FDD11) - a brief disability disaggregation instrument that countries can use.

**Results:**

The study demonstrated that FDD11 is a valid and reliable tool. Unidimensionality of the scale produced by each calibration was supported by the factor analysis performed. The analysis indicated good fit of the items, and targeting of the items was deemed to be sufficient. The person separation index was 0.82, indicating good reliability of the final scale.

**Conclusion:**

FDD11 provides a good opportunity to researchers and governments to capture good quality disability data and to disaggregate existing data by disability. The tool can facilitate low- and middle-income countries in their efforts to develop evidenced-based policies to address any barriers faced by persons with disabilities, to monitor the implementation of the Convention on the Rights of Persons with Disabilities and the Sustainable Development Goals, and to take stock of the challenges that still remain.

## Background

Disability results from the interaction between an individual’s health condition (such as cerebral palsy or depression) and personal and environmental factors including negative attitudes, inaccessible transportation or public buildings, and limited social support [1]. Today, over 1 billion people or about 15% of the global population have significant disability, and this number is increasing [2]. While persons with disabilities are a very diverse group, they commonly have less access to health services, education, and work opportunities and are more likely to live in poverty compared to those without disabilities [2].

Disaggregated data is fundamental to identify the inequities that persons with disabilities face globally as well as their magnitude. Disaggregating data consists of breaking down data by segments, e.g. disability, in order to understand in detail patterns that can be masked by larger aggregate data. More specifically, disaggregated data can highlight where inequities exist and bring out further specifics that are essential for effective programme planning and inclusive policies [3]. Recognizing its importance, the 2030 Sustainable Development Agenda affirms that data should be disaggregated by disability in addition to age, gender, income, ethnicity, race, geographic location and other characteristics in order to achieve the concept of “leaving no one behind” [4]. Such disaggregation is fundamental for countries to be able to develop evidenced-based policies to monitor the implementation of the Convention on the Rights of Persons with Disabilities (CRPD), the Sustainable Development Goals (SDGs), to measure progress towards national targets, and to take stock of the challenges that still remain.

Despite the importance of disaggregating data and the increased global interest in collecting disability data over the past years, very few countries report good quality, comparable and consistent data [3]. This is due to several factors. Firstly, the way disability data is collected varies significantly across countries [5]. These distinct approaches lead to major differences in the prevalence estimates of disability, making any comparability efforts almost impossible. Secondly, even when disability data exists in countries, it is not disaggregated in an accessible manner [3]. An example of the lack of country reporting on disability data is the fact that since the beginning of the Covid-19 pandemic, UK has been the only country to report Covid-19 health data disaggregated by disability, revealing that those with disability have been disproportionately affected by the crisis, both in terms of morbidity and mortality [6, 7].

This scarcity of good quality disability data has contributed to a lack of political will to address the situation and challenges that persons with disability face. In fact, a recent UN Sustainable Development Goals Report [8] stated that the absence of sound disaggregated data by disability has limited the global understanding of the discrimination and exclusion that persons with disabilities experience on a daily basis. The current paper proposes a way forward to overcome the lack of good quality and comparable data on disability by introducing a valid and reliable disability disaggregation instrument that countries can integrate into existing tools. The objective of this study is to demonstrate the psychometric properties of the WHO Functioning and Disability Disaggregation Tool (FDD11) - a short 11-question instrument derived from the WHO Model Disability Survey (MDS) [9, 10] – a standalone WHO household survey that has been validated and broadly implemented in countries. The items included in FDD11 have been carefully and systematically selected from the MDS to capture the experience of disability by assessing difficulties when undertaking various activities associated with a health problem.

## Methods

### Development of FDD11

The FDD11 was derived as a standalone instrument for disaggregation purposes from the MDS. More specifically, the 11 questions constituted a separate module on intrinsic capacity that was part of the brief version of the MDS. Details on the development of the brief MDS are reported elsewhere [11], but a short summary is provided below.

WHO developed the full version of the MDS in 2014 to answer the global call for collection of comparable and valid disability data. The MDS represented an evolution in the concept of disability measurement assessing three aspects to fully describe the experience of disability – 1) capacity, defined as the synthesis of all intrinsic physical and mental capacities of a person, determined by their health condition or impairments; 2) functioning, defined as the outcome of the interaction between the individual’s capacity and features of the environment; and 3) environmental factors that affect the individual’s lived experience of disability [11].

From as early as 2016, countries requested a brief version of the MDS to be used as a module that could be integrated into existing surveys to allow for monitoring disability prevalence or collecting information on environmental barriers for evidence-informed policymaking [9, 10]. The brief MDS was developed through an expert consensus process and analytical work [12]. To select a brief set of questions that will capture a similar amount of information as the full questionnaire, firstly, experts in functioning and disability measurement selected questions based on social and cultural universality, relevance to the WHO International Classification of Functioning, Disability and Health (ICF), and statistical criteria. Secondly, the reliability of the expert selection was tested using Generalized Partial Credit Model (GPCM) and Bayesian models [12]. The final model for the Brief MDS drew upon the same three core modules of the full MDS (environmental factors, functioning, and capacity), yielding excellent reliability and explaining a high proportion of the variance of the scores from the long version of the questionnaire.

The capacity module in particular included 11 questions, which constitute the FDD11 disaggregation tool. FDD11 was created as a separate instrument for three main reasons – 1) to allow for a quick integration in existing surveys, 2) to capture functioning information needed for disaggregating data by disability level, and most importantly – 3) to allow for a quick, sound and valid disaggregation by disability. The tool measures functioning and disability by assessing difficulties in seeing; hearing; walking or climbing steps; remembering or concentrating; washing all over or dressing; sleeping; doing household tasks; joining community activities, such as festivities, religious or other activities; feeling sad, low, worried or anxious; getting along with close people including family and friends; and bodily aches and pain. The response options provided for each question vary from 1 “none” to 5 “extreme” difficulties.

### Design and sample

The current psychometric study uses pooled secondary data from cross-sectional studies carried out between 2014 and 2019 in Afghanistan; Adamawa, Cameroon; Chile; Costa Rica; India; Tajikistan; Laos; Balochistan, Pakistan; Philippines; Qatar; and Sri Lanka, with a total sample of 66,800 adults interviewed. The analysis includes samples from countries that have conducted the full MDS and from those who have applied the brief version of the MDS. In terms of data collection, nine surveys were nationally representative and two were regional implementations. A representative sample of the population of the country or region was drawn, and one household member was randomly selected to answer to the individual questionnaire in a face-to-face interview. All participants were informed of the purpose and rationale of the study upon participation. Ethical approvals for each of the country implementations of the MDS were obtained from the respective national health authorities. No filters were applied to pre-select a population with disability or health conditions. In the case of India, Tajikistan and Laos, the Brief MDS was implemented as a module of the Gallup World Poll. Individuals participating in the surveys were generally adults aged 18 or more, but some countries took the decision to include people between 15 and 17 years of age in the sample. Table 1 shows the demographic characteristics of the sample.

**Table 1:** Demographic characteristics of the sample from the implementation of the Model Disability Survey in Afghanistan; Adamawa, Cameroon; Chile; Costa Rica; India; Tajikistan; Laos; Balochistan, Pakistan; Philippines; Qatar; and Sri Lanka in the period 2014–2019

Demographics	Value	Number	Percentage
**Sex**	Male	30,816	46.1
	Female	35,714	53.5
	N/A	270	0.4
**Age**	15–47	43,734	65.5
	48–63	14,667	22
	64+	8087	12.1
	N/A	312	0.5
**Education**	Elementary or less	29,147	43.6
	Secondary	22,425	33.6
	Tertiary	14,400	21.6
	N/A	828	1.2
	**Total**	**66,800**	**100**

### Data analysis

Psychometric properties of the FDD11 were assessed using Rasch Analysis [13], more specifically with the Partial Credit Model for polytomous data [14]. The Rasch Model for polytomous data is a modern test theory approach that can be used to test the assumption that there is unidimensional latent construct that can be measured reliably with the data collection tool. It is expected that the items of a measurement scale match the population they assess; the items of a scale are expected to address the continuum of the latent trait being measured to determine levels of that trait. In this case, the latent construct we are measuring is disability. Both the persons (i.e., respondents) and items can be located on the same latent continuum under the Rasch Model: for persons, the Rasch model provides estimates that describe their individual level of disability, and for items estimates are found that describe their difficulty. The Rasch Model implies a hierarchical construct between the items wherein it is expected that a person who reports difficulty on an item also reports difficulty on all other items before it on the latent continuum. Conceptually, this may seem counterintuitive for measurement of disability, because it is entirely possible that a person can have health conditions or impairments along one domain (e.g. hearing) and not another (e.g. mobility). However, it is well known that a person experiencing disability is very likely to experience other comorbidities [15, 16], so the hierarchical construct of the Rasch Model is reasonable.

When applied in an iterative process, the Rasch analysis can also serve as a method to identify the best way to use one’s data to generate a valid and reliable metric of the latent continuum. The model is run on the ordinal dataset, assumptions of the Rasch Model are checked, the data is adjusted in order to improve model fit, and the model is run again. The process repeats until adequate adherence to the Rasch Model assumptions is found.

The following assumptions of the Rasch Model were assessed at each iteration of the model:

1. *Independence of the items*: Item independence was assessed using the correlation matrix of Rasch residuals [17]. Items were considered independent if the residual correlation was less than 0.2 plus the mean of the residual correlations [18].
2. *Unidimensionality of the scale*: Bi-factor analysis was applied to assess unidimensionality [19]. As part of the bi-factor analyses process, polychoric correlation coefficients for ordered-category data were calculated, and parallel analysis was performed that determined how many factors should be retained in the bi-factor analysis. For unidimensionality to be met, all questions in the instrument have to load higher in the general factor (that loads all items) than in the specific factors [20, 21].
3. *Ordering of the thresholds*: Threshold ordering was studied based on the threshold locations for each item. “Thresholds” refer to the point between Likert scale response options on the latent continuum where a respondent is equally likely to choose two adjacent response options [22]. In this case, each question had 5 response options (ranging from 1 = no difficulty to 5 = extreme difficulty) and therefore there were 4 thresholds whose ordering was assessed. In case items’ thresholds were disordered, response options for those items had to be collapsed [22].
4. *Item and person fit*: Item fit was assessed based on the infit mean square statistics, having been shown that they are relatively independent of sample size for polytomous data, and that a range between 0.7 and 1.3 can be used to indicate good item fit [19]. Person fit was assessed also using infit mean square statistics, using a more generous range of > 2 to indicate poor fit [23].
5. *Targeting of the scale and reliability of the model*: Targeting was explored by comparing the distribution of persons’ abilities and item thresholds along the latent trait continuum, whereas reliability was studied with the Person Separation Index r_ß_, ranging between zero and 1, where 1 indicated perfect reproducibility of person placements [24].
6. *Differential item functioning*: Lastly, differential item functioning (DIF) was examined to indicate whether items work the same way, irrespective of the country the survey was implemented in. Mitigation of DIF was considered for each item, balancing the severity of the DIF with the impact of mitigation strategies on the face validity of the questionnaire and its ability to uncover true differences in the disability experienced across different cultural contexts.

In addition, by using Rasch analysis, we can condense a broad range of questions about different functioning domains rated using an ordinal scale from 1 (no problems) to 5 (extreme problems) into a continuum, or metrical scale, of disability ranging from zero (no disability) to 100 (severe disability). In this way, ratings of each question are not simply added to create a sum score per person, because it is known that extreme problems in different life areas might indicate different overall levels of disability, as demonstrated elsewhere [16]. Simply adding up scores across areas assumes that these problems are indeed comparable. Rasch analyses is applied because it provides a robust methodology to create a valid interval scale with true metrical properties and calculate individual scores, which is needed considering that questions have different “weights”, in terms of level of disability that they capture. In this sense, even if the original questions provide a range from 1 to 5, the Rasch analysis transforms this into a continuum on which cut-offs are applied to differentiate between levels of disability.

More details on Rasch analysis methodology can be found elsewhere [16].

Respondents were retained in the analysis if they had two or fewer missing values from the survey items. As a final step, persons’ abilities that were obtained on a logit scale were linearly transformed to an intuitive scale ranging from zero (no disability) to 100 (extreme disability). Then cut-offs were applied to the scale to identify four groups: no disability, mild disability, moderate disability, and severe disability. The cut-offs that were applied included 1) the mean score minus 1 standard deviation of the score; 2) the mean score; and 3) the mean score plus 1 standard deviation of the score. All data analyses were performed in R v4.0.0 [25], and the package used for Rasch Analysis was eRm v1.0–1 (https://cran.r-project.org/web/packages/eRm/index.html).

## Results

The initial iteration of the Rasch Model was run with 12 questions. After removing respondents with 2 or more missing values in these 12 questions, 47,997 respondents were retained in the analysis. The item regarding feeling “sad, low, depressed, worried nervous or anxious” is included in several country implementations as two separate items (“sad, low, or depressed” and “worried, nervous or anxious”). These two items showed high residual correlation (r = 0.38), indicating that they were not independent items. This high residual correlation supported the formation of a single combined item (i.e., a testlet). Therefore, in all subsequent iterations of the Rasch model these two items were combined into one item by summing the responses of these two items and recoding response options to a 5-point scale. The high correlation between the two items also supported the inclusion of only one item along this emotion domain in the final FDD11 questionnaire.

The Rasch Model was then run again on the FDD11 data using this new testlet. In the following iteration, all items except the depression/anxiety testlet, “bodily aches”, and “walking or climbing steps” exhibited disordered thresholds. The model was further iteratively applied with the goal of resolving the disordered thresholds. At each iteration, response options for the items were collapsed, attempting to make as minimum a change to the response options as possible to achieve threshold ordering. Table 2 shows the recode strategy used in the final model.

**Table 2:** Threshold recoding strategy used in final Rasch model applied on the data from the implementation of the Model Disability Survey in Afghanistan; Adamawa, Cameroon; Chile; Costa Rica; India; Tajikistan; Laos; Balochistan, Pakistan; Philippines; Qatar; and Sri Lanka in the period 2014–2019

Number	Question	Original response options	Recoded response options
Q01	Do you have difficulty seeing, even if wearing glasses?	01234	01233
Q02	Do you have difficulty hearing, even if using a hearing aid?	01234	01122
Q03	Do you have difficulty walking or climbing steps?	01234	01233
Q04	Do you have difficulty remembering or concentrating?	01234	01233
Q05	Do you have difficulty (with self-care such as) washing all over or dressing?	01234	01122
Q06	How much difficulty do you have sleeping because of your health?	01234	01122
Q07	How much difficulty do you have doing household tasks because of your health?	01234	01122
Q08	Because of your health, how much difficulty do you have with joining community activities, such as festivities, religious or other activities?	01234	01122
Q09	To what extent do you feel sad, low or depressed because of your health? To what extent do you feel worried, nervous or anxious because of your health?	012345678	011223344
Q10	Because of your health, how much difficulty do you have getting along with people who are close to you, including your family and friends?	01234	01233
Q11	How much bodily aches or pains do you have?	01234	no recoding performed

One pair of items “difficulty doing household tasks” and “joining community activities” had residual correlation above the threshold of 0.11 (r = 0.13). Given the residual correlation being only slightly above the threshold and the conceptual differences covered by each question, it was deemed that this correlation was still at an acceptable level.

**Fig. 1:**
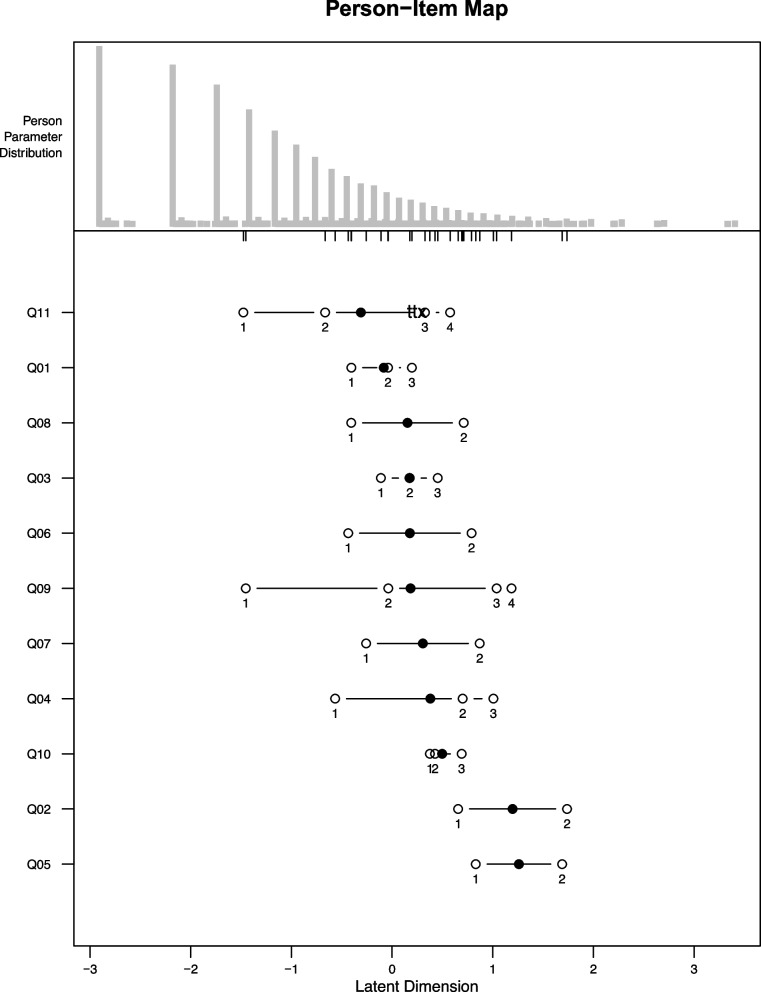
Person Item Map of the Distribution of persons’ abilities, items’ locations (black circles) and items’ thresholds (white circles) on the latent continuum, based on the data from the implementation of the Model Disability Survey in Afghanistan; Adamawa, Cameroon; Chile; Costa Rica; India; Tajikistan; Laos; Balochistan, Pakistan; Philippines; Qatar; and Sri Lanka in the period 2014–2019

Unidimensionality of the scale produced by each calibration was supported by the factor analysis performed: for the final scale, parallel analysis indicated the number of factors in the bi-factor analysis should equal 10, and the subsequent bi-factor analysis showed that the loadings on the general factor was higher than on specific factors. Figure 1 shows the distribution of persons’ abilities, items’ locations and items’ thresholds on the latent continuum.

Table 3 shows the infit statistics, locations and thresholds of items. All infit except for the item related to “seeing” fell between 0.7 and 1.3, indicating good fit. Infit for the seeing item was 1.43. Because this value is not far from the upper threshold of 1.3 and because of the importance of including an item on vision for face validity of the FDD11 instrument, the “seeing” item was retained in the final questionnaire.

**Table 3:** Item characteristics from the final Rasch model: infit statistics, item location, and item thresholds with threshold standard errors, based on the data from the implementation of the Model Disability Survey in Afghanistan; Adamawa, Cameroon; Chile; Costa Rica; India; Tajikistan; Laos; Balochistan, Pakistan; Philippines; Qatar; and Sri Lanka in the period 2014–2019

Number	Question	Infit	Location	Threshold 1 (SE)	Threshold 2 (SE)	Threshold 3 (SE)	Threshold 4 (SE)
Q01	Do you have difficulty seeing, even if wearing glasses?	1.43	−0.08	− 0.4 (0.01)	− 0.04 (0.02)	0.2 (0.02)	NA
Q02	Do you have difficulty hearing, even if using a hearing aid?	0.92	1.2	0.66 (0.01)	1.74 (0.03)	NA	NA
Q03	Do you have difficulty walking or climbing steps?	0.81	0.17	−0.11 (0.01)	0.18 (0.02)	0.45 (0.03)	NA
Q04	Do you have difficulty remembering or concentrating?	0.89	0.38	−0.57 (0.01)	0.7 (0.02)	1.01 (0.03)	NA
Q05	Do you have difficulty (with self-care such as) washing all over or dressing?	0.71	1.26	0.83 (0.01)	1.69 (0.03)	NA	NA
Q06	How much difficulty do you have sleeping because of your health?	0.83	0.18	−0.44 (0.01)	0.79 (0.02)	NA	NA
Q07	How much difficulty do you have doing household tasks because of your health?	0.76	0.31	−0.26 (0.01)	0.87 (0.02)	NA	NA
Q08	Because of your health, how much difficulty do you have with joining community activities, such as festivities, religious or other activities?	0.88	0.15	−0.41 (0.01)	0.71 (0.02)	NA	NA
Q09	To what extent do you feel sad, low or depressed because of your health? To what extent do you feel worried, nervous or anxious because of your health?	1.01	0.18	−1.45 (0.01)	−0.04 (0.01)	1.04 (0.02)	1.19 (0.04)
Q10	Because of your health, how much difficulty do you have getting along with people who are close to you, including your family and friends?	0.99	0.5	0.38 (0.01)	0.43 (0.02)	0.69 (0.03)	NA
Q11	How much bodily aches or pains do you have?	0.96	−0.31	−1.48 (0.01)	−0.67 (0.01)	0.33 (0.02)	0.58 (0.03)

Person fit was also assessed using infit mean square statistics. Of the 47,997 respondents assessed, 2771 (5.8%) had an infit mean squared value above 2.0. Given that in general one would expect approximately 5% of misfit by chance [26], and the skewed nature of the sample with respect to disability, it was not deemed serious enough misfit to remove any respondents further from the analysis.

Table 4 shows results relevant to targeting. Targeting of the items was deemed to be sufficient: the survey instrument targets health issues, but most of the population reports no health issues, as expected because the sample comes from the general population. Therefore the targeting shows that a high proportion of the population has lower functioning problems than what the scale addresses. The person separation index was 0.82, indicating good reliability of the final scale.

**Table 4:** Mean, SD and range of item and person locations on the latent continuum, mean and SD of residuals, and person separation index, based on the data from the implementation of the Model Disability Survey in Afghanistan; Adamawa, Cameroon; Chile; Costa Rica; India; Tajikistan; Laos; Balochistan, Pakistan; Philippines; Qatar; and Sri Lanka in the period 2014–2019

	Location mean	Location SD	Location range	Residuals mean	Residuals SD
Items	0.2863	0.7788	−1.48 to 1.74	0.019	0.0077
Persons	−1.2733	1.0822	−2.91 to 3.41	0.5776	0.2227
Person separation index (PSI)	0.82				

All items showed DIF with respect to the country in which the MDS was implemented, with F-test *p*-values (df = 10) below 0.001 in all cases, indicating that items may have been understood differently depending on where the survey was implemented. Table 5 demonstrates the DIF results for country of MDS implementation.

**Table 5:** Differential item functioning (DIF) results for country of MDS implementation in Afghanistan; Adamawa, Cameroon; Chile; Costa Rica; India; Tajikistan; Laos; Balochistan, Pakistan; Philippines; Qatar; and Sri Lanka in the period 2014–2019

Number	Question	df	F value	*P*-value
Q01	Do you have difficulty seeing, even if wearing glasses?	10	1567.62	< 0.0001
Q02	Do you have difficulty hearing, even if using a hearing aid?	10	51.53	< 0.0001
Q03	Do you have difficulty walking or climbing steps?	10	146.38	< 0.0001
Q04	Do you have difficulty remembering or concentrating?	10	166.13	< 0.0001
Q05	Do you have difficulty (with self-care such as) washing all over or dressing?	10	312.14	< 0.0001
Q06	How much difficulty do you have sleeping because of your health?	10	92.09	< 0.0001
Q07	How much difficulty do you have doing household tasks because of your health?	10	314.17	< 0.0001
Q08	Because of your health, how much difficulty do you have with joining community activities, such as festivities, religious or other activities?	10	283.86	< 0.0001
Q09	To what extent do you feel sad, low or depressed because of your health? To what extent do you feel worried, nervous or anxious because of your health?	10	407.24	< 0.0001
Q10	Because of your health, how much difficulty do you have getting along with people who are close to you, including your family and friends?	10	409.01	< 0.0001
Q11	How much bodily aches or pains do you have?	10	140.08	< 0.0001

The DIF evidenced by the FDD11 items is logical and is not surprising, given that notions of what constitutes “good health” vary across cultural contexts [27]. For example, Q07 of the FDD11 asks respondents to rate their difficulty performing household tasks. Typical methods for household tasks, like washing clothes, could vary widely between a lower-middle income country setting like Adamawa, Cameroon vs a high income setting like Chile (https://datahelpdesk.worldbank.org/knowledgebase/articles/906519-world-bank-country-and-lending-groups). DIF may also be observed because of true differences in the underlying level of disability experienced across country populations. Because the DIF results are plausible and cultural differences are expected, fairness of the tool across cultural contexts is not necessarily sacrificed [28].

In order to justify modifying or removing an item on the basis of DIF, it is usually necessary to uncover the mediating process that links the variable in question (country implementation) to the measured outcome (items of the FDD11) [29]. In this circumstance we have plausible theories for the link between the two, but concrete evidence of the linkage requires further study. Resolving DIF regarding country implementation would result in a disability score that attenuates differences between countries, potentially reducing effect sizes and masking true underlying differences in the disability levels experienced between populations, which is not desirable in situations where the FDD11 is used to analyze differences between countries. Furthermore, we lack reasonable methods to resolve DIF without undermining the FDD11 tool completely. Two main options for resolving DIF exist: removing items or splitting them into separate items along the factor where DIF is observed. Deleting all items was clearly not possible. Splitting items was also not feasible because then a separate scale would need to be generated for each implementation. We decided to not attempt to resolve DIF along the dimension of country implementation, given that the DIF results are plausible and do not sacrifice fairness of the tool, and due to the lack of a reasonable method for resolving it.

Persons’ abilities were linearly transformed into a scale ranging from 0 to 100 and also mapped to the sum score that results when the recoded response options are added together. The 0–100 scale was then disaggregated into four disability levels: no, mild, moderate and severe disability. These categories were determined from cut-offs based on the mean and standard deviation of the transformed scale: the first cut-off is the mean minus the standard deviation (4.3); the second cut-off is the mean (22.6); the third cut-off is the mean plus the standard deviation (40.8). The number of disability categories used was supported by the PSI of 0.82, which is above the accepted threshold for assessment of groups of individuals [30]. Table 6 shows the range of scores on the 0–100 scale and the sum score of each disability level.

**Table 6:** Ranges of scores that define disability levels of no, mild, moderate and severe disability for the data from the implementation of the Model Disability Survey in Afghanistan; Adamawa, Cameroon; Chile; Costa Rica; India; Tajikistan; Laos; Balochistan, Pakistan; Philippines; Qatar; and Sri Lanka in the period 2014–2019

Disability level	Transformed scale range	Sum score range
No	0–4.3	0–0
Mild	4.3–22.6	1–2
Moderate	22.6–40.8	3–8
Severe	40.8–100	9–30

Table 7 shows the assignment of sum scores to persons’ abilities and the transformed metric scores on a scale ranging from 0 to 100.

**Table 7:** Assignment of sum scores, persons’ abilities with standard errors and transformed scores on a 0–100 scale of the FDD11 metric for the data from the implementation of the Model Disability Survey in Afghanistan; Adamawa, Cameroon; Chile; Costa Rica; India; Tajikistan; Laos; Balochistan, Pakistan; Philippines; Qatar; and Sri Lanka in the period 2014–2019

Sum score*	Persons’ abilities (SE)	Transformed scale	Disability level
0	−3.71	0	No
1	−2.91 (1.02)	10.12	Mild
2	−2.18 (0.731)	19.36	Mild
3	−1.74 (0.605)	24.93	Moderate
4	−1.42 (0.531)	28.99	Moderate
5	−1.17 (0.481)	32.23	Moderate
6	−0.952 (0.445)	34.94	Moderate
7	−0.766 (0.418)	37.29	Moderate
8	−0.601 (0.397)	39.39	Moderate
9	−0.45 (0.38)	41.30	Severe
10	−0.31 (0.368)	43.08	Severe
11	−0.179 (0.358)	44.74	Severe
12	−0.0536 (0.351)	46.33	Severe
13	0.0674 (0.346)	47.87	Severe
14	0.186 (0.343)	49.37	Severe
15	0.303 (0.341)	50.85	Severe
16	0.419 (0.342)	52.33	Severe
17	0.537 (0.344)	53.82	Severe
18	0.657 (0.349)	55.34	Severe
19	0.781 (0.355)	56.91	Severe
20	0.91 (0.364)	58.55	Severe
21	1.05 (0.375)	60.28	Severe
22	1.19 (0.39)	62.14	Severe
23	1.35 (0.41)	64.16	Severe
24	1.53 (0.435)	66.42	Severe
25	1.73 (0.47)	69.01	Severe
26	1.98 (0.518)	72.09	Severe
27	2.28 (0.591)	75.95	Severe
28	2.7 (0.716)	81.27	Severe
29	3.41 (1)	90.20	Severe
30	4.18	100	Severe

## Discussion

This study demonstrates the good psychometric properties of the WHO FDD11 – a short instrument that can be integrated into existing surveys to allow for data disaggregation by disability. The Rasch analysis applied in the study proved that the tool can successfully measure disability as a construct. The instrument provides an excellent opportunity to researchers and governments to capture good quality disability data and to disaggregate existing data by disability.

Disaggregation of indicators by disability is fundamental for understanding in depth and improving the situation of persons with disabilities. For example, data on girls and women with disabilities helps us understand the elevated discrimination and inequities they experience. Covid-19 mortality data from the UK disaggregated by disability showed that women were more disproportionately affected by the virus compared to men, and that most of the deaths occurred in long-term care institutions [6, 7]. This information is useful for the government to strengthen their emergency preparedness and response plans to be inclusive of the specific needs of women with disabilities in a pandemic as well as to the living conditions in institutions. Furthermore, because of studies that have disaggregated data by disability we know that women with disabilities are more vulnerable to abuse and intimate partner violence [31–33]. Understanding how abuse affects persons with disabilities, and particular women and girls, is key to designing prevention strategies to ensure autonomy and reduce risk of harm.

Currently, there are several distinct approaches used to collect and disaggregate data by disability. Some countries or research groups use a single item in their censuses or national surveys to identify those with disability, e.g. “Do you have a disability?” [34, 35]. Others use a medical diagnosis as a definition of disability, counting those who have preselected health conditions or impairments associated with high levels of disability (e.g. traumatic brain injury or deafness) [34]. A third group of instruments include those assessing functioning limitations referring to a set of difficulties that people experience in undertaking specific activities like walking, seeing, or hearing. These outcome measures are different from the ones using a medical diagnosis because they address what a person can or cannot do, as opposed to the reason why they cannot do it [36–38]. The Washington Group Questions (WGQ) is a screening instrument used by many countries that takes this approach [39].

There are well-known issues with single-item screeners and medical approaches to disability data collection. Using a single item to define who has disability can lead to underreporting because the term “disability” is often associated with an assumption of a severe condition, and people might not report their status correctly [34]. Medical diagnosis can also lead to under-reporting as those without access to health services may not have been diagnosed by a healthcare professional, and also people with the same health conditions can have different levels of functioning limitations [36–38].

Although several tools are currently used for disaggregation, studies reporting on their development, specificity, sensitivity, and psychometric properties are not common. The studies that do so, reveal some inconsistencies and limitations. For example, a study comparing the WGQ to clinical impairment screening using data from Cameroon and India showed that the WGQ correctly identified only 33% of participants in Cameroon and 45% in India as disabled, i.e. the sensitivity of the questions ranged between 30 and 45% [40]. Similarly, the Australian Bureau of Statistics (ABS) implemented the WGQ on a sample of respondents from the 2015 ABS Survey of Disability, Ageing and Carers (SDAC) [41]. Results showed that only 27% of people with disability in the SDAC were correctly identified as having a disability by the WGQ. Another study by Sabariego et al. [42] raised concerns about the use of screening instruments that might lead to imprecise rates and misclassify persons with mild to moderate disability as non-disabled. The current study shows that the good psychometric properties of the FDD11 are a reflection of a careful and systematic development process that is the basis for achieving a brief but valid assessment of disability.

There are several reasons why the FDD11 overcomes the limitations of other instruments and stands as a valid and reliable tool that countries can apply for data disaggregation by disability. Firstly, the tool captures a wider set of domains of functioning. Secondly, the tool is very brief and can be administered quickly. Thirdly, unlike other instruments, FDD11 provides disability prevalence by severity levels. This follows WHO concept of disability as a universal experience and a continuum that can range from no to severe disability [1]. Having a clear idea of the number and percentage of people with mild or moderate levels of disability is as important as identifying those with severe disability. This information can help and support creating policies and strategies on disability that address the needs of every person with disability and also prevent those having mild or moderate disability from becoming severe cases. Lastly, a user-friendly excel file for analysis of data is currently under development by WHO to facilitate countries’ use of the tool. The excel file will include an algorithm that allows data owners to input the data collected through the FDD11 and automatically obtain the distribution of disability in the population with percentage of persons with no, mild, moderate and severe disability. In addition, the file will convert the raw numerical data into a metric of disability, which can be used for data disaggregation purposes by sex, age or any relevant variables in further analyses.

This study has to be seen in the light of certain limitations. Firstly, the metrics of targeting indicate that the survey items most directly measure persons in the mid-range of the disability continuum, and less directly measures persons with high levels of disability. However, because a main purpose of the tool is to identify who experiences disability and who does not—as opposed to gathering detailed insights within each group—the targeting was deemed sufficient for this purpose. This is a result of the data being assessed in a general population and not focused exclusively on persons pre-screened to experience significant disability. The targeting results points to the usefulness of the FDD11 as a disaggregation tool, particularly between persons with higher levels of disability vs lower levels; however the tool is not best-suited to allow for deeper analyses within the population experiencing severe disability or for making individual disability determination assessments. Secondly, the FDD11 exhibits severe DIF along the dimension of country implementation, indicating that questions may be understood differently across cultural contexts and that survey results between countries may not be directly comparable. DIF is a well-known measurement issue [43], but on its own does not necessarily indicate bias in the tool, if the DIF is plausible and explainable (https://datahelpdesk.worldbank.org/knowledgebase/articles/906519-world-bank-country-and-lending-groups) as it is in the case of the FDD11. Additionally, any potential issues with DIF may be attenuated by the FDD11’s use of four categories as opposed to specific scores to measure disability. However, it may be advisable that further calibration be done for implementations of the FDD11 that are meant to be directly comparable across cultural contexts [44, 45], for instance similarly to the FAO Food Insecurity Experience Scale [46]. Thirdly, it must be recognized that attitudinal, social and built environmental factors all contribute significantly to persons’ disability experience, and in order to function as a rapid measurement tool, the FDD11 does not assess these complex dimensions. The full WHO MDS goes into great depth on environmental factors and can be used as an alternative for studies whose main purpose is measuring how the environment contributes to disability.

## Conclusion

In conclusion, the current study provides evidence for a valid and reliable tool developed by WHO - FDD11 – which can be integrated into existing surveys to allow for quick and coherent data disaggregation by disability. The tool can facilitate countries in their efforts to develop evidenced-based policies to address any barriers faced by persons with disabilities, to monitor the implementation of the Convention on the Rights of Persons with Disabilities (CRPD) and the Sustainable Development Goals (SDGs), and to take stock of the challenges that still remain.

## Data Availability

Data are available from the authors upon request.

## Abbreviations

WHO: World Health Organization
FDD11: Functioning and disability disaggregation tool
ICF: International classification of functioning, disability and health
